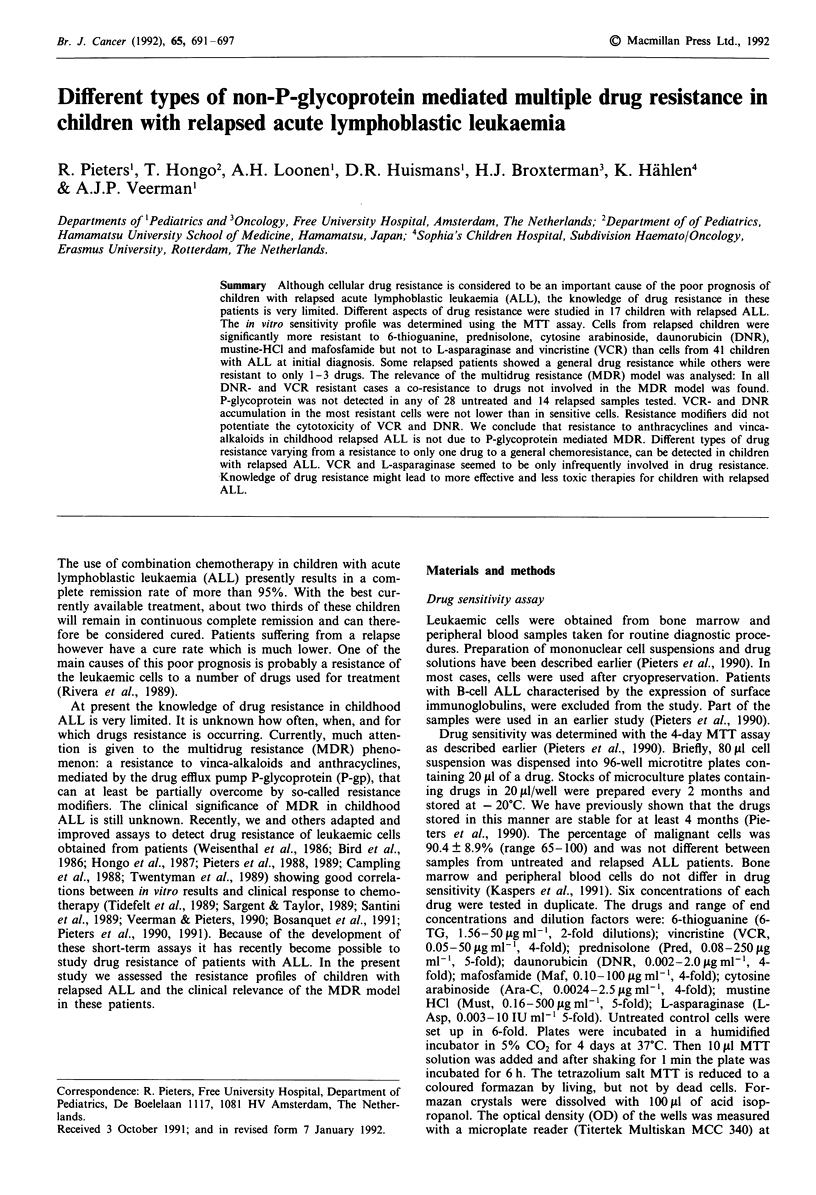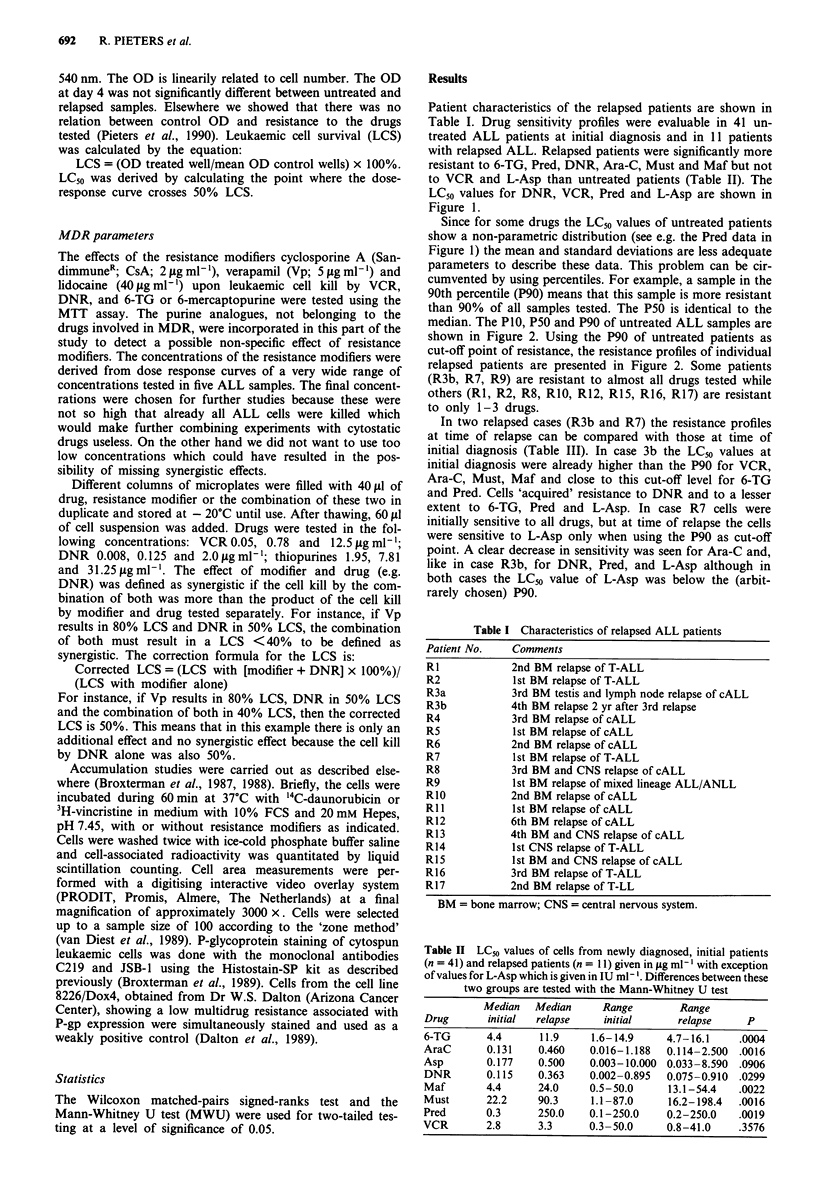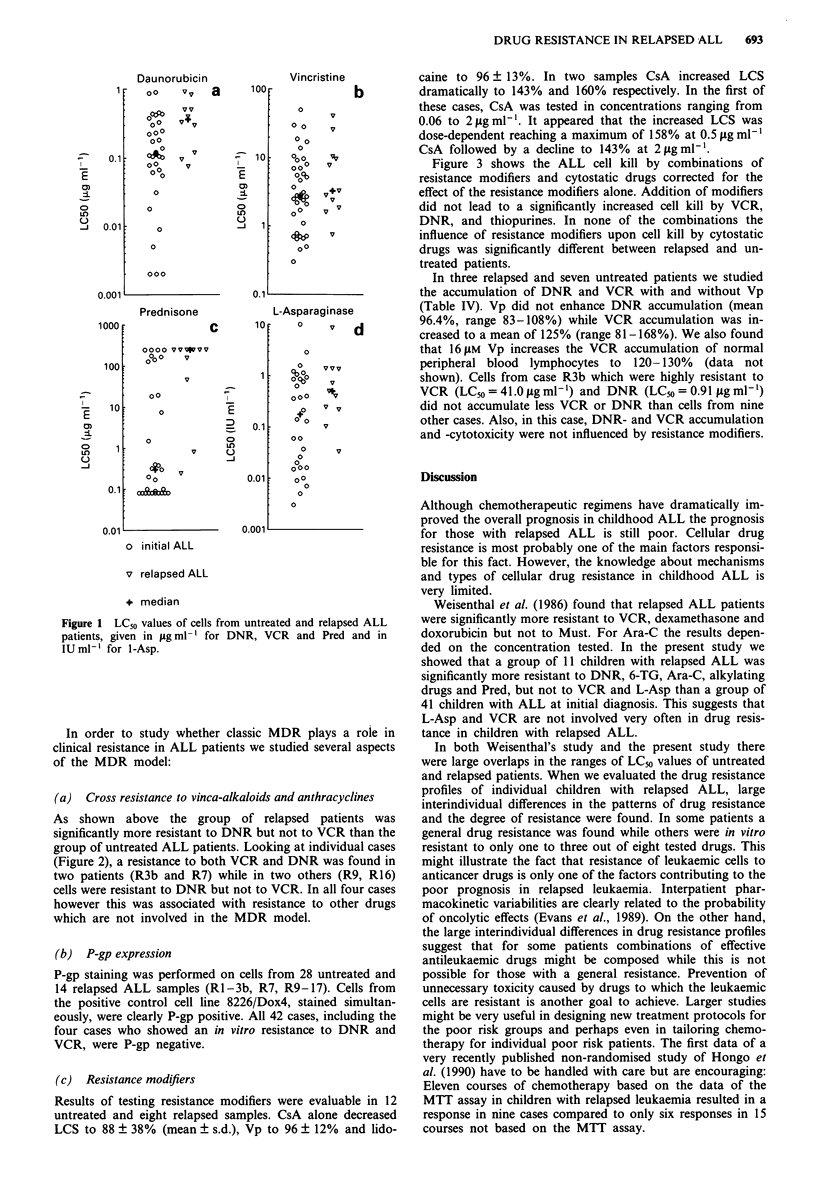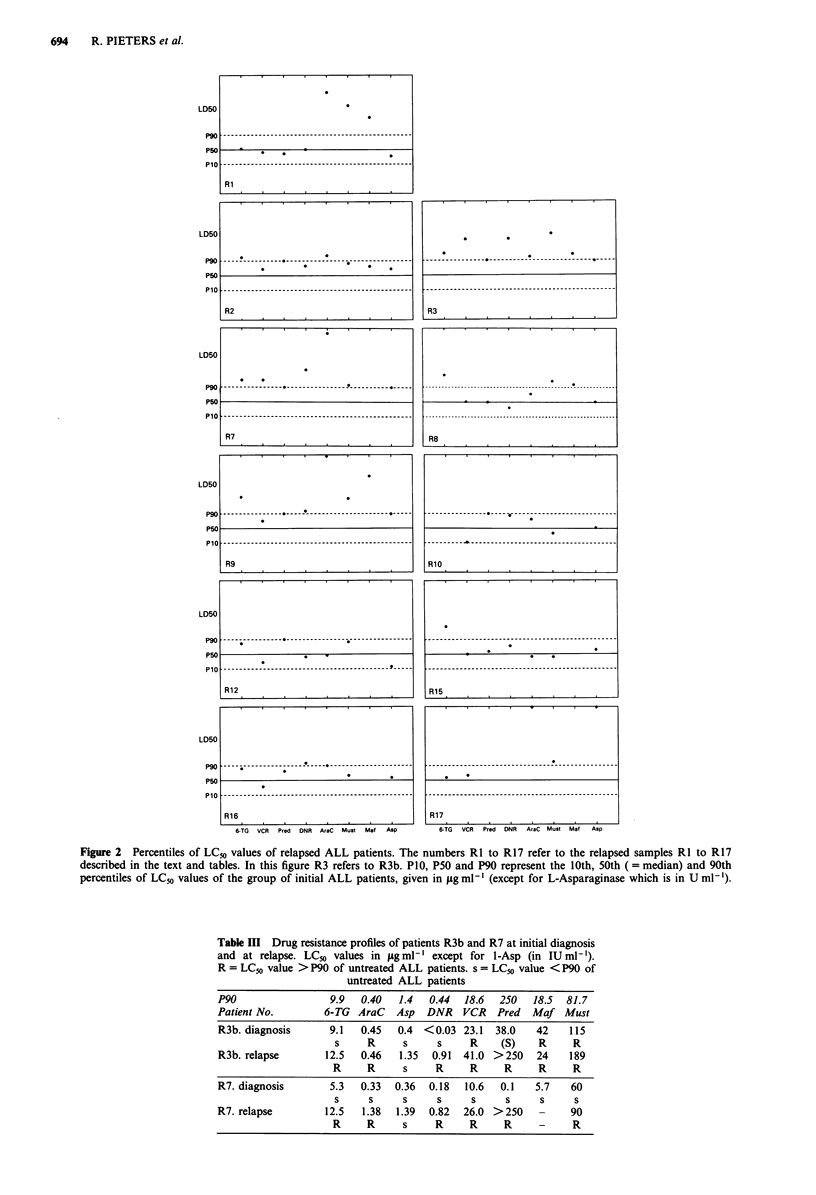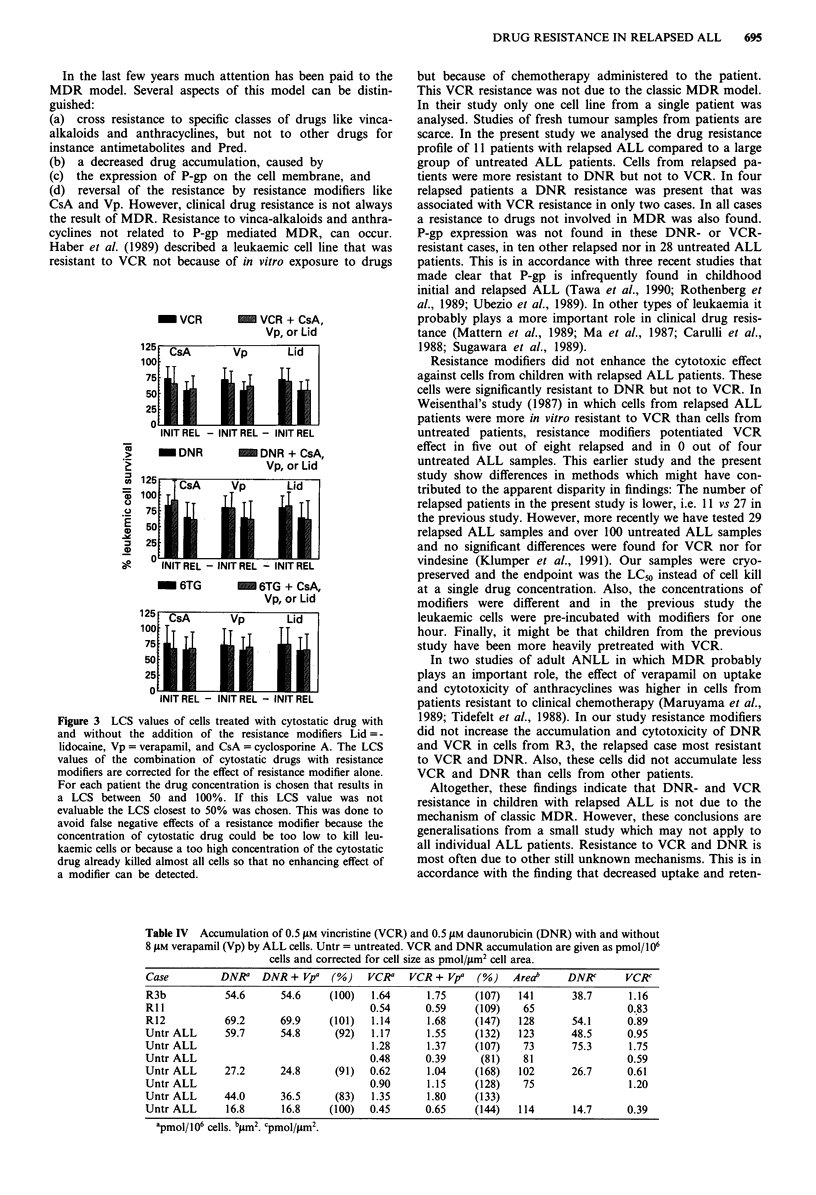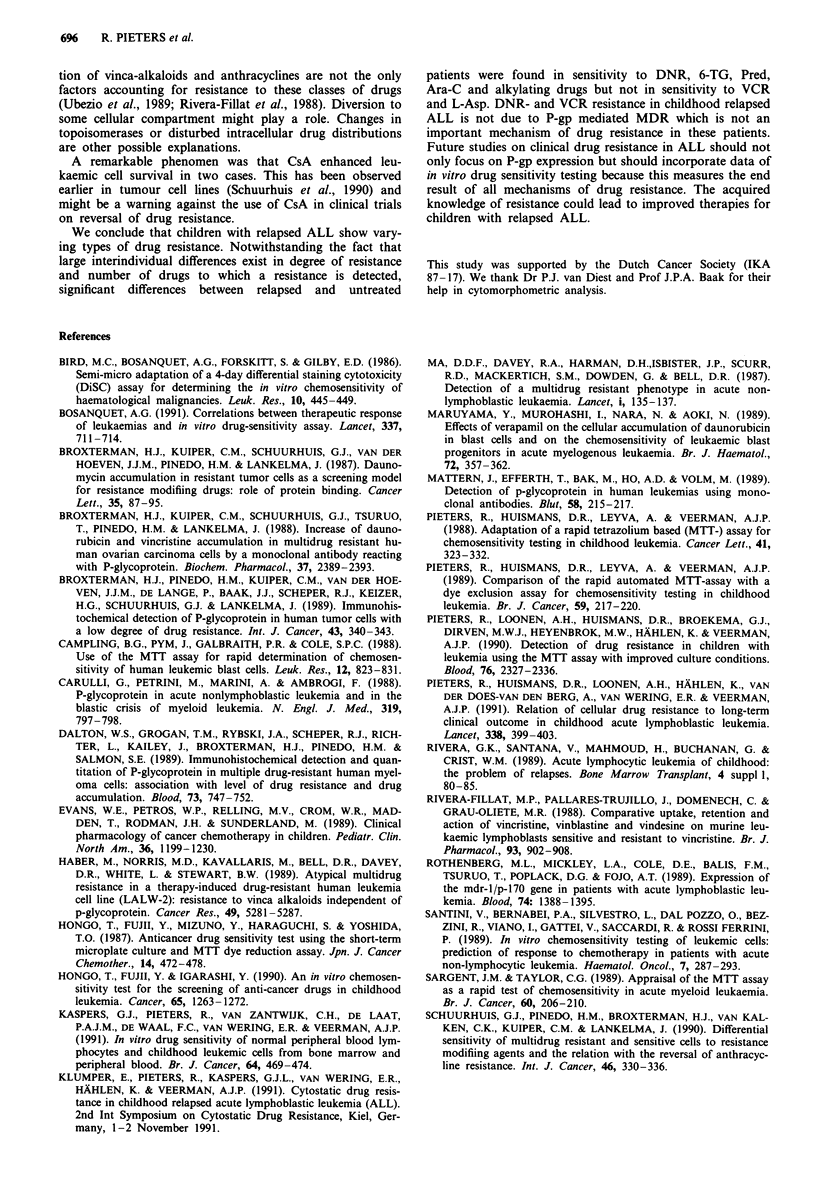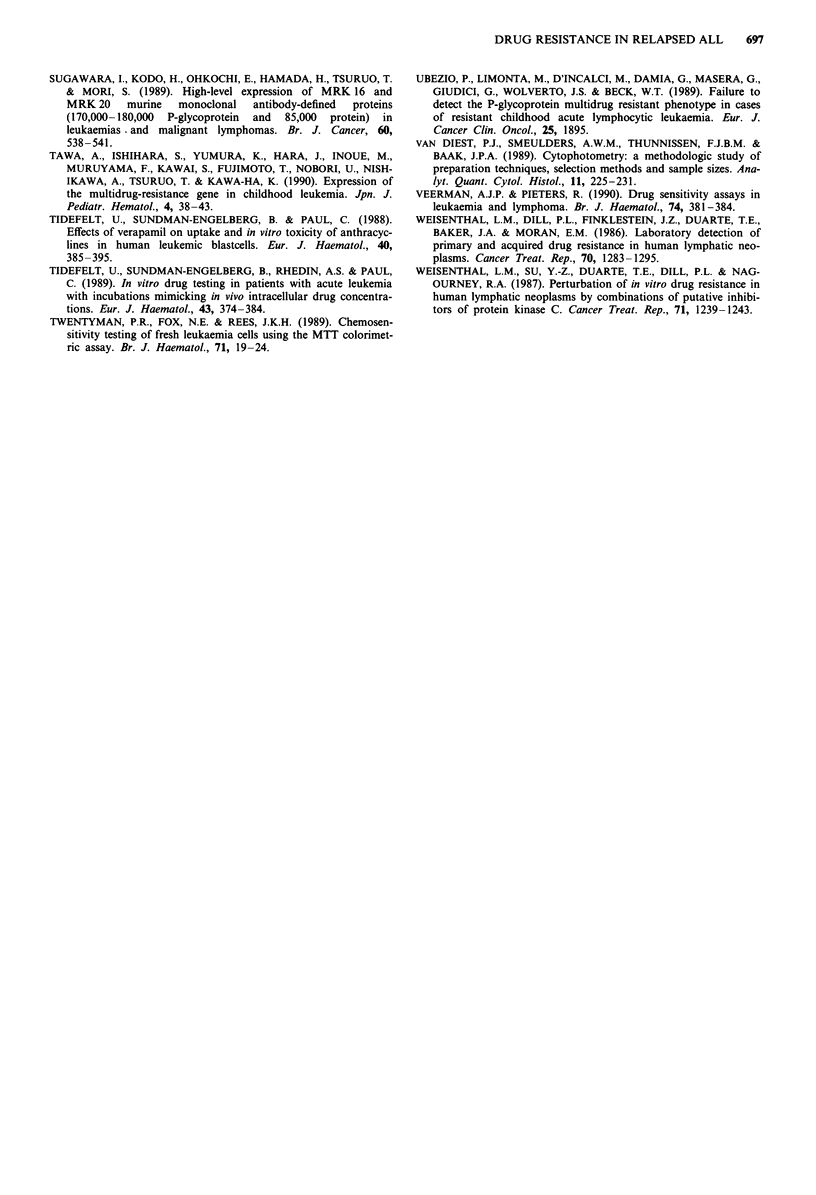# Different types of non-P-glycoprotein mediated multiple drug resistance in children with relapsed acute lymphoblastic leukaemia.

**DOI:** 10.1038/bjc.1992.146

**Published:** 1992-05

**Authors:** R. Pieters, T. Hongo, A. H. Loonen, D. R. Huismans, H. J. Broxterman, K. Hählen, A. J. Veerman

**Affiliations:** Department of Pediatrics, Free University Hospital, Amsterdam, The Netherlands.

## Abstract

**Images:**


 © Macmillan Press Ltd., 1992

### Different types of non-P-glycoprotein mediated multiple drug resistance in children with relapsed acute lymphoblastic leukaemia

R. Pieters[1], T. Hongo[2], A.H. Loonen[1], D.R. Huismans[1], H.J. Broxterman[3], K. Hählen[4] & A.J.P. Veerman[1]

*Departments of [1]Pediatrics and [3]Oncology, Free University Hospital, Amsterdam, The Netherlands; [2]Department of of Pediatrics, Hamamatsu University School of Medicine, Hamamatsu, Japan; [4]Sophia's Children Hospital, Subdivision Haemato/Oncology, Erasmus University, Rotterdam, The Netherlands.*

**Summary** Although cellular drug resistance is considered to be an important cause of the poor prognosis of children with relapsed acute lymphoblastic leukaemia (ALL), the knowledge of drug resistance in these patients is very limited. Different aspects of drug resistance were studied in 17 children with relapsed ALL. The *in vitro* sensitivity profile was determined using the MTT assay. Cells from relapsed children were significantly more resistant to 6-thioguanine, prednisolone, cytosine arabinoside, daunorubicin (DNR), mustine-HCl and mafosfamide but not to L-asparaginase and vincristine (VCR) than cells from 41 children with ALL at initial diagnosis. Some relapsed patients showed a general drug resistance while others were resistant to only 1-3 drugs. The relevance of the multidrug resistance (MDR) model was analysed: In all DNR- and VCR resistant cases a co-resistance to drugs not involved in the MDR model was found. P-glycoprotein was not detected in any of 28 untreated and 14 relapsed samples tested. VCR- and DNR accumulation in the most resistant cells were not lower than in sensitive cells. Resistance modifiers did not potentiate the cytotoxicity of VCR and DNR. We conclude that resistance to anthracyclines and vinca-alkaloids in childhood relapsed ALL is not due to P-glycoprotein mediated MDR. Different types of drug resistance varying from a resistance to only one drug to a general chemoresistance, can be detected in children with relapsed ALL. VCR and L-asparaginase seemed to be only infrequently involved in drug resistance. Knowledge of drug resistance might lead to more effective and less toxic therapies for children with relapsed ALL.

The use of combination chemotherapy in children with acute lymphoblastic leukaemia (ALL) presently results in a complete remission rate of more than 95%. With the best currently available treatment, about two thirds of these children will remain in continuous complete remission and can therefore be considered cured. Patients suffering from a relapse however have a cure rate which is much lower. One of the main causes of this poor prognosis is probably a resistance of the leukaemic cells to a number of drugs used for treatment (Rivera *et al.*, 1989).

At present the knowledge of drug resistance in childhood ALL is very limited. It is unknown how often, when, and for which drugs resistance is occurring. Currently, much attention is given to the multidrug resistance (MDR) phenomenon: a resistance to vinca-alkaloids and anthracyclines, mediated by the drug efflux pump P-glycoprotein (P-gp), that can at least be partially overcome by so-called resistance modifiers. The clinical significance of MDR in childhood ALL is still unknown. Recently, we and others adapted and improved assays to detect drug resistance of leukaemic cells obtained from patients (Weisenthal *et al.*, 1986; Bird *et al.*, 1986; Hongo *et al.*, 1987; Pieters *et al.*, 1988, 1989; Campling *et al.*, 1988; Twentyman *et al.*, 1989) showing good correlations between *in vitro* results and clinical response to chemotherapy (Tidefelt *et al.*, 1989; Sargent & Taylor, 1989; Santini *et al.*, 1989; Veerman & Pieters, 1990; Bosanquet *et al.*, 1991; Pieters *et al.*, 1990, 1991). Because of the development of these short-term assays it has recently become possible to study drug resistance of patients with ALL. In the present study we assessed the resistance profiles of children with relapsed ALL and the clinical relevance of the MDR model in these patients.

Correspondence: R. Pieters, Free University Hospital, Department of Pediatrics, De Boelelaan 1117, 1081 HV Amsterdam, The Netherlands.
Received 3 October 1991; and in revised form 7 January 1992.

### Materials and methods

### *Drug sensitivity assay*

Leukaemic cells were obtained from bone marrow and peripheral blood samples taken for routine diagnostic procedures. Preparation of mononuclear cell suspensions and drug solutions have been described earlier (Pieters *et al.*, 1990). In most cases, cells were used after cryopreservation. Patients with B-cell ALL characterised by the expression of surface immunoglobulins, were excluded from the study. Part of the samples were used in an earlier study (Pieters *et al.*, 1990).

Drug sensitivity was determined with the 4-day MTT assay as described earlier (Pieters *et al.*, 1990). Briefly, 80 µl cell suspension was dispensed into 96-well microtitre plates containing 20 µl of a drug. Stocks of microculture plates containing drugs in 20 µl/well were prepared every 2 months and stored at − 20°C. We have previously shown that the drugs stored in this manner are stable for at least 4 months (Pieters *et al.*, 1990). The percentage of malignant cells was 90.4 ± 8.9% (range 65-100) and was not different between samples from untreated and relapsed ALL patients. Bone marrow and peripheral blood cells do not differ in drug sensitivity (Kaspers *et al.*, 1991). Six concentrations of each drug were tested in duplicate. The drugs and range of end concentrations and dilution factors were: 6-thioguanine (6-TG, 1.56-50 µg ml$^{-1}$, 2-fold dilutions); vincristine (VCR, 0.05-50 µg ml$^{-1}$, 4-fold); prednisolone (Pred, 0.08-250 µg ml$^{-1}$, 5-fold); daunorubicin (DNR, 0.002-2.0 µg ml$^{-1}$, 4-fold); mafosfamide (Maf, 0.10-100 µg ml$^{-1}$, 4-fold); cytosine arabinoside (Ara-C, 0.0024-2.5 µg ml$^{-1}$, 4-fold); mustine HCl (Must, 0.16-500 µg ml$^{-1}$, 5-fold); L-asparaginase (L-Asp, 0.003-10 IU ml$^{-1}$ 5-fold). Untreated control cells were set up in 6-fold. Plates were incubated in a humidified incubator in 5% $CO_2$ for 4 days at 37°C. Then 10 µl MTT solution was added and after shaking for 1 min the plate was incubated for 6 h. The tetrazolium salt MTT is reduced to a coloured formazan by living, but not by dead cells. Formazan crystals were dissolved with 100 µl of acid isopropanol. The optical density (OD) of the wells was measured with a microplate reader (Titertek Multiskan MCC 340) at

540 nm. The OD is linearly related to cell number. The OD at day 4 was not significantly different between untreated and relapsed samples. Elsewhere we showed that there was no relation between control OD and resistance to the drugs tested (Pieters *et al.*, 1990). Leukaemic cell survival (LCS) was calculated by the equation:

LCS = (OD treated well/mean OD control wells) × 100%. $LC_{50}$ was derived by calculating the point where the dose-response curve crosses 50% LCS.

### *MDR parameters*

The effects of the resistance modifiers cyclosporine A (Sandimmune[R]; CsA; 2 μg ml$^{-1}$), verapamil (Vp; 5 μg ml$^{-1}$) and lidocaine (40 μg ml$^{-1}$) upon leukaemic cell kill by VCR, DNR, and 6-TG or 6-mercaptopurine were tested using the MTT assay. The purine analogues, not belonging to the drugs involved in MDR, were incorporated in this part of the study to detect a possible non-specific effect of resistance modifiers. The concentrations of the resistance modifiers were derived from dose response curves of a very wide range of concentrations tested in five ALL samples. The final concentrations were chosen for further studies because these were not so high that already all ALL cells were killed which would make further combining experiments with cytostatic drugs useless. On the other hand we did not want to use too low concentrations which could have resulted in the possibility of missing synergistic effects.

Different columns of microplates were filled with 40 μl of drug, resistance modifier or the combination of these two in duplicate and stored at − 20°C until use. After thawing, 60 μl of cell suspension was added. Drugs were tested in the following concentrations: VCR 0.05, 0.78 and 12.5 μg ml$^{-1}$; DNR 0.008, 0.125 and 2.0 μg ml$^{-1}$; thiopurines 1.95, 7.81 and 31.25 μg ml$^{-1}$. The effect of modifier and drug (e.g. DNR) was defined as synergistic if the cell kill by the combination of both was more than the product of the cell kill by modifier and drug tested separately. For instance, if Vp results in 80% LCS and DNR in 50% LCS, the combination of both must result in a LCS <40% to be defined as synergistic. The correction formula for the LCS is:

Corrected LCS = (LCS with [modifier + DNR] × 100%)/(LCS with modifier alone)

For instance, if Vp results in 80% LCS, DNR in 50% LCS and the combination of both in 40% LCS, then the corrected LCS is 50%. This means that in this example there is only an additional effect and no synergistic effect because the cell kill by DNR alone was also 50%.

Accumulation studies were carried out as described elsewhere (Broxterman *et al.*, 1987, 1988). Briefly, the cells were incubated during 60 min at 37°C with $^{14}$C-daunorubicin or $^{3}$H-vincristine in medium with 10% FCS and 20 mM Hepes, pH 7.45, with or without resistance modifiers as indicated. Cells were washed twice with ice-cold phosphate buffer saline and cell-associated radioactivity was quantitated by liquid scintillation counting. Cell area measurements were performed with a digitising interactive video overlay system (PRODIT, Promis, Almere, The Netherlands) at a final magnification of approximately 3000 ×. Cells were selected up to a sample size of 100 according to the 'zone method' (van Diest *et al.*, 1989). P-glycoprotein staining of cytospun leukaemic cells was done with the monoclonal antibodies C219 and JSB-1 using the Histostain-SP kit as described previously (Broxterman *et al.*, 1989). Cells from the cell line 8226/Dox4, obtained from Dr W.S. Dalton (Arizona Cancer Center), showing a low multidrug resistance associated with P-gp expression were simultaneously stained and used as a weakly positive control (Dalton *et al.*, 1989).

### *Statistics*

The Wilcoxon matched-pairs signed-ranks test and the Mann-Whitney U test (MWU) were used for two-tailed testing at a level of significance of 0.05.

### Results

Patient characteristics of the relapsed patients are shown in Table I. Drug sensitivity profiles were evaluable in 41 untreated ALL patients at initial diagnosis and in 11 patients with relapsed ALL. Relapsed patients were significantly more resistant to 6-TG, Pred, DNR, Ara-C, Must and Maf but not to VCR and L-Asp than untreated patients (Table II). The $LC_{50}$ values for DNR, VCR, Pred and L-Asp are shown in Figure 1.

Since for some drugs the $LC_{50}$ values of untreated patients show a non-parametric distribution (see e.g. the Pred data in Figure 1) the mean and standard deviations are less adequate parameters to describe these data. This problem can be circumvented by using percentiles. For example, a sample in the 90th percentile (P90) means that this sample is more resistant than 90% of all samples tested. The P50 is identical to the median. The P10, P50 and P90 of untreated ALL samples are shown in Figure 2. Using the P90 of untreated patients as cut-off point of resistance, the resistance profiles of individual relapsed patients are presented in Figure 2. Some patients (R3b, R7, R9) are resistant to almost all drugs tested while others (R1, R2, R8, R10, R12, R15, R16, R17) are resistant to only 1–3 drugs.

In two relapsed cases (R3b and R7) the resistance profiles at time of relapse can be compared with those at time of initial diagnosis (Table III). In case 3b the $LC_{50}$ values at initial diagnosis were already higher than the P90 for VCR, Ara-C, Must, Maf and close to this cut-off level for 6-TG and Pred. Cells 'acquired' resistance to DNR and to a lesser extent to 6-TG, Pred and L-Asp. In case R7 cells were initially sensitive to all drugs, but at time of relapse the cells were sensitive to L-Asp only when using the P90 as cut-off point. A clear decrease in sensitivity was seen for Ara-C and, like in case R3b, for DNR, Pred, and L-Asp although in both cases the $LC_{50}$ value of L-Asp was below the (arbitrarely chosen) P90.

**Table I** Characteristics of relapsed ALL patients

| *Patient No.* | *Comments* |
|---|---|
| R1 | 2nd BM relapse of T-ALL |
| R2 | 1st BM relapse of T-ALL |
| R3a | 3rd BM testis and lymph node relapse of cALL |
| R3b | 4th BM relapse 2 yr after 3rd relapse |
| R4 | 3rd BM relapse of cALL |
| R5 | 1st BM relapse of cALL |
| R6 | 2nd BM relapse of cALL |
| R7 | 1st BM relapse of T-ALL |
| R8 | 3rd BM and CNS relapse of cALL |
| R9 | 1st BM relapse of mixed lineage ALL/ANLL |
| R10 | 2nd BM relapse of cALL |
| R11 | 1st BM relapse of cALL |
| R12 | 6th BM relapse of cALL |
| R13 | 4th BM and CNS relapse of cALL |
| R14 | 1st CNS relapse of T-ALL |
| R15 | 1st BM and CNS relapse of cALL |
| R16 | 3rd BM relapse of T-ALL |
| R17 | 2nd BM relapse of T-LL |

BM = bone marrow; CNS = central nervous system.

**Table II** $LC_{50}$ values of cells from newly diagnosed, initial patients ($n$ = 41) and relapsed patients ($n$ = 11) given in μg ml$^{-1}$ with exception of values for L-Asp which is given in IU ml$^{-1}$. Differences between these two groups are tested with the Mann-Whitney U test

| *Drug* | *Median initial* | *Median relapse* | *Range initial* | *Range relapse* | *P* |
|---|---|---|---|---|---|
| 6-TG | 4.4 | 11.9 | 1.6–14.9 | 4.7–16.1 | .0004 |
| AraC | 0.131 | 0.460 | 0.016–1.188 | 0.114–2.500 | .0016 |
| Asp | 0.177 | 0.500 | 0.003–10.000 | 0.033–8.590 | .0906 |
| DNR | 0.115 | 0.363 | 0.002–0.895 | 0.075–0.910 | .0299 |
| Maf | 4.4 | 24.0 | 0.5–50.0 | 13.1–54.4 | .0022 |
| Must | 22.2 | 90.3 | 1.1–87.0 | 16.2–198.4 | .0016 |
| Pred | 0.3 | 250.0 | 0.1–250.0 | 0.2–250.0 | .0019 |
| VCR | 2.8 | 3.3 | 0.3–50.0 | 0.8–41.0 | .3576 |

**Figure 1** $LC_{50}$ values of cells from untreated and relapsed ALL patients, given in µg ml$^{-1}$ for DNR, VCR and Pred and in IU ml$^{-1}$ for l-Asp.

In order to study whether classic MDR plays a role in clinical resistance in ALL patients we studied several aspects of the MDR model:

*(a) Cross resistance to vinca-alkaloids and anthracyclines*

As shown above the group of relapsed patients was significantly more resistant to DNR but not to VCR than the group of untreated ALL patients. Looking at individual cases (Figure 2), a resistance to both VCR and DNR was found in two patients (R3b and R7) while in two others (R9, R16) cells were resistant to DNR but not to VCR. In all four cases however this was associated with resistance to other drugs which are not involved in the MDR model.

*(b) P-gp expression*

P-gp staining was performed on cells from 28 untreated and 14 relapsed ALL samples (R1–3b, R7, R9–17). Cells from the positive control cell line 8226/Dox4, stained simultaneously, were clearly P-gp positive. All 42 cases, including the four cases who showed an *in vitro* resistance to DNR and VCR, were P-gp negative.

*(c) Resistance modifiers*

Results of testing resistance modifiers were evaluable in 12 untreated and eight relapsed samples. CsA alone decreased LCS to 88 ± 38% (mean ± s.d.), Vp to 96 ± 12% and lidocaine to 96 ± 13%. In two samples CsA increased LCS dramatically to 143% and 160% respectively. In the first of these cases, CsA was tested in concentrations ranging from 0.06 to 2 µg ml$^{-1}$. It appeared that the increased LCS was dose-dependent reaching a maximum of 158% at 0.5 µg ml$^{-1}$ CsA followed by a decline to 143% at 2 µg ml$^{-1}$.

Figure 3 shows the ALL cell kill by combinations of resistance modifiers and cytostatic drugs corrected for the effect of the resistance modifiers alone. Addition of modifiers did not lead to a significantly increased cell kill by VCR, DNR, and thiopurines. In none of the combinations the influence of resistance modifiers upon cell kill by cytostatic drugs was significantly different between relapsed and untreated patients.

In three relapsed and seven untreated patients we studied the accumulation of DNR and VCR with and without Vp (Table IV). Vp did not enhance DNR accumulation (mean 96.4%, range 83–108%) while VCR accumulation was increased to a mean of 125% (range 81–168%). We also found that 16 µM Vp increases the VCR accumulation of normal peripheral blood lymphocytes to 120–130% (data not shown). Cells from case R3b which were highly resistant to VCR ($LC_{50}$ = 41.0 µg ml$^{-1}$) and DNR ($LC_{50}$ = 0.91 µg ml$^{-1}$) did not accumulate less VCR or DNR than cells from nine other cases. Also, in this case, DNR- and VCR accumulation and -cytotoxicity were not influenced by resistance modifiers.

### Discussion

Although chemotherapeutic regimens have dramatically improved the overall prognosis in childhood ALL the prognosis for those with relapsed ALL is still poor. Cellular drug resistance is most probably one of the main factors responsible for this fact. However, the knowledge about mechanisms and types of cellular drug resistance in childhood ALL is very limited.

Weisenthal *et al.* (1986) found that relapsed ALL patients were significantly more resistant to VCR, dexamethasone and doxorubicin but not to Must. For Ara-C the results depended on the concentration tested. In the present study we showed that a group of 11 children with relapsed ALL was significantly more resistant to DNR, 6-TG, Ara-C, alkylating drugs and Pred, but not to VCR and L-Asp than a group of 41 children with ALL at initial diagnosis. This suggests that L-Asp and VCR are not involved very often in drug resistance in children with relapsed ALL.

In both Weisenthal's study and the present study there were large overlaps in the ranges of $LC_{50}$ values of untreated and relapsed patients. When we evaluated the drug resistance profiles of individual children with relapsed ALL, large interindividual differences in the patterns of drug resistance and the degree of resistance were found. In some patients a general drug resistance was found while others were *in vitro* resistant to only one to three out of eight tested drugs. This might illustrate the fact that resistance of leukaemic cells to anticancer drugs is only one of the factors contributing to the poor prognosis in relapsed leukaemia. Interpatient pharmacokinetic variabilities are clearly related to the probability of oncolytic effects (Evans *et al.*, 1989). On the other hand, the large interindividual differences in drug resistance profiles suggest that for some patients combinations of effective antileukaemic drugs might be composed while this is not possible for those with a general resistance. Prevention of unnecessary toxicity caused by drugs to which the leukaemic cells are resistant is another goal to achieve. Larger studies might be very useful in designing new treatment protocols for the poor risk groups and perhaps even in tailoring chemotherapy for individual poor risk patients. The first data of a very recently published non-randomised study of Hongo *et al.* (1990) have to be handled with care but are encouraging: Eleven courses of chemotherapy based on the data of the MTT assay in children with relapsed leukaemia resulted in a response in nine cases compared to only six responses in 15 courses not based on the MTT assay.

**Figure 2** Percentiles of $LC_{50}$ values of relapsed ALL patients. The numbers R1 to R17 refer to the relapsed samples R1 to R17 described in the text and tables. In this figure R3 refers to R3b. P10, P50 and P90 represent the 10th, 50th ( = median) and 90th percentiles of $LC_{50}$ values of the group of initial ALL patients, given in $\mu g\,ml^{-1}$ (except for L-Asparaginase which is in $U\,ml^{-1}$).

**Table III** Drug resistance profiles of patients R3b and R7 at initial diagnosis and at relapse. $LC_{50}$ values in $\mu g\,ml^{-1}$ except for 1-Asp (in $IU\,ml^{-1}$). R = $LC_{50}$ value >P90 of untreated ALL patients. s = $LC_{50}$ value <P90 of untreated ALL patients

| *P90* | *9.9* | *0.40* | *1.4* | *0.44* | *18.6* | *250* | *18.5* | *81.7* |
|---|---|---|---|---|---|---|---|---|
| *Patient No.* | *6-TG* | *AraC* | *Asp* | *DNR* | *VCR* | *Pred* | *Maf* | *Must* |
| R3b. diagnosis | 9.1 | 0.45 | 0.4 | <0.03 | 23.1 | 38.0 | 42 | 115 |
| | s | R | s | s | R | (S) | R | R |
| R3b. relapse | 12.5 | 0.46 | 1.35 | 0.91 | 41.0 | >250 | 24 | 189 |
| | R | R | s | R | R | R | R | R |
| R7. diagnosis | 5.3 | 0.33 | 0.36 | 0.18 | 10.6 | 0.1 | 5.7 | 60 |
| | s | s | s | s | s | s | s | s |
| R7. relapse | 12.5 | 1.38 | 1.39 | 0.82 | 26.0 | >250 | – | 90 |
| | R | R | s | R | R | R | – | R |

In the last few years much attention has been paid to the MDR model. Several aspects of this model can be distinguished:

(a) cross resistance to specific classes of drugs like vinca-alkaloids and anthracyclines, but not to other drugs for instance antimetabolites and Pred.
(b) a decreased drug accumulation, caused by
(c) the expression of P-gp on the cell membrane, and
(d) reversal of the resistance by resistance modifiers like CsA and Vp. However, clinical drug resistance is not always the result of MDR. Resistance to vinca-alkaloids and anthracyclines not related to P-gp mediated MDR, can occur. Haber *et al.* (1989) described a leukaemic cell line that was resistant to VCR not because of *in vitro* exposure to drugs

**Figure 3** LCS values of cells treated with cytostatic drug with and without the addition of the resistance modifiers Lid = lidocaine, Vp = verapamil, and CsA = cyclosporine A. The LCS values of the combination of cytostatic drugs with resistance modifiers are corrected for the effect of resistance modifier alone. For each patient the drug concentration is chosen that results in a LCS between 50 and 100%. If this LCS value was not evaluable the LCS closest to 50% was chosen. This was done to avoid false negative effects of a resistance modifier because the concentration of cytostatic drug could be too low to kill leukaemic cells or because a too high concentration of the cytostatic drug already killed almost all cells so that no enhancing effect of a modifier can be detected.

but because of chemotherapy administered to the patient. This VCR resistance was not due to the classic MDR model. In their study only one cell line from a single patient was analysed. Studies of fresh tumour samples from patients are scarce. In the present study we analysed the drug resistance profile of 11 patients with relapsed ALL compared to a large group of untreated ALL patients. Cells from relapsed patients were more resistant to DNR but not to VCR. In four relapsed patients a DNR resistance was present that was associated with VCR resistance in only two cases. In all cases a resistance to drugs not involved in MDR was also found. P-gp expression was not found in these DNR- or VCR-resistant cases, in ten other relapsed nor in 28 untreated ALL patients. This is in accordance with three recent studies that made clear that P-gp is infrequently found in childhood initial and relapsed ALL (Tawa *et al.*, 1990; Rothenberg *et al.*, 1989; Ubezio *et al.*, 1989). In other types of leukaemia it probably plays a more important role in clinical drug resistance (Mattern *et al.*, 1989; Ma *et al.*, 1987; Carulli *et al.*, 1988; Sugawara *et al.*, 1989).

Resistance modifiers did not enhance the cytotoxic effect against cells from children with relapsed ALL patients. These cells were significantly resistant to DNR but not to VCR. In Weisenthal's study (1987) in which cells from relapsed ALL patients were more *in vitro* resistant to VCR than cells from untreated patients, resistance modifiers potentiated VCR effect in five out of eight relapsed and in 0 out of four untreated ALL samples. This earlier study and the present study show differences in methods which might have contributed to the apparent disparity in findings: The number of relapsed patients in the present study is lower, i.e. 11 *vs* 27 in the previous study. However, more recently we have tested 29 relapsed ALL samples and over 100 untreated ALL samples and no significant differences were found for VCR nor for vindesine (Klumper *et al.*, 1991). Our samples were cryopreserved and the endpoint was the $LC_{50}$ instead of cell kill at a single drug concentration. Also, the concentrations of modifiers were different and in the previous study the leukaemic cells were pre-incubated with modifiers for one hour. Finally, it might be that children from the previous study have been more heavily pretreated with VCR.

In two studies of adult ANLL in which MDR probably plays an important role, the effect of verapamil on uptake and cytotoxicity of anthracyclines was higher in cells from patients resistant to clinical chemotherapy (Maruyama *et al.*, 1989; Tidefelt *et al.*, 1988). In our study resistance modifiers did not increase the accumulation and cytotoxicity of DNR and VCR in cells from R3, the relapsed case most resistant to VCR and DNR. Also, these cells did not accumulate less VCR and DNR than cells from other patients.

Altogether, these findings indicate that DNR- and VCR resistance in children with relapsed ALL is not due to the mechanism of classic MDR. However, these conclusions are generalisations from a small study which may not apply to all individual ALL patients. Resistance to VCR and DNR is most often due to other still unknown mechanisms. This is in accordance with the finding that decreased uptake and reten-

**Table IV** Accumulation of 0.5 μM vincristine (VCR) and 0.5 μM daunorubicin (DNR) with and without 8 μM verapamil (Vp) by ALL cells. Untr = untreated. VCR and DNR accumulation are given as pmol/$10^6$ cells and corrected for cell size as pmol/$\mu m^2$ cell area.

| *Case* | *DNR*[a] | *DNR + Vp*[a] | *(%)* | *VCR*[a] | *VCR + Vp*[a] | *(%)* | *Area*[b] | *DNR*[c] | *VCR*[c] |
|---|---|---|---|---|---|---|---|---|---|
| R3b | 54.6 | 54.6 | (100) | 1.64 | 1.75 | (107) | 141 | 38.7 | 1.16 |
| R11 | | | | 0.54 | 0.59 | (109) | 65 | | 0.83 |
| R12 | 69.2 | 69.9 | (101) | 1.14 | 1.68 | (147) | 128 | 54.1 | 0.89 |
| Untr ALL | 59.7 | 54.8 | (92) | 1.17 | 1.55 | (132) | 123 | 48.5 | 0.95 |
| Untr ALL | | | | 1.28 | 1.37 | (107) | 73 | 75.3 | 1.75 |
| Untr ALL | | | | 0.48 | 0.39 | (81) | 81 | | 0.59 |
| Untr ALL | 27.2 | 24.8 | (91) | 0.62 | 1.04 | (168) | 102 | 26.7 | 0.61 |
| Untr ALL | | | | 0.90 | 1.15 | (128) | 75 | | 1.20 |
| Untr ALL | 44.0 | 36.5 | (83) | 1.35 | 1.80 | (133) | | | |
| Untr ALL | 16.8 | 16.8 | (100) | 0.45 | 0.65 | (144) | 114 | 14.7 | 0.39 |

[a]pmol/$10^6$ cells. [b]$\mu m^2$. [c]pmol/$\mu m^2$.

tion of vinca-alkaloids and anthracyclines are not the only factors accounting for resistance to these classes of drugs (Ubezio et al., 1989; Rivera-Fillat et al., 1988). Diversion to some cellular compartment might play a role. Changes in topoisomerases or disturbed intracellular drug distributions are other possible explanations.

A remarkable phenomen was that CsA enhanced leukaemic cell survival in two cases. This has been observed earlier in tumour cell lines (Schuurhuis et al., 1990) and might be a warning against the use of CsA in clinical trials on reversal of drug resistance.

We conclude that children with relapsed ALL show varying types of drug resistance. Notwithstanding the fact that large interindividual differences exist in degree of resistance and number of drugs to which a resistance is detected, significant differences between relapsed and untreated patients were found in sensitivity to DNR, 6-TG, Pred, Ara-C and alkylating drugs but not in sensitivity to VCR and L-Asp. DNR- and VCR resistance in childhood relapsed ALL is not due to P-gp mediated MDR which is not an important mechanism of drug resistance in these patients. Future studies on clinical drug resistance in ALL should not only focus on P-gp expression but should incorporate data of *in vitro* drug sensitivity testing because this measures the end result of all mechanisms of drug resistance. The acquired knowledge of resistance could lead to improved therapies for children with relapsed ALL.

This study was supported by the Dutch Cancer Society (IKA 87-17). We thank Dr P.J. van Diest and Prof J.P.A. Baak for their help in cytomorphometric analysis.

### References

BIRD, M.C., BOSANQUET, A.G., FORSKITT, S. & GILBY, E.D. (1986). Semi-micro adaptation of a 4-day differential staining cytotoxicity (DiSC) assay for determining the *in vitro* chemosensitivity of haematological malignancies. *Leuk. Res.*, **10**, 445-449.

BOSANQUET, A.G. (1991). Correlations between therapeutic response of leukaemias and *in vitro* drug-sensitivity assay. *Lancet*, **337**, 711-714.

BROXTERMAN, H.J., KUIPER, C.M., SCHUURHUIS, G.J., VAN DER HOEVEN, J.J.M., PINEDO, H.M. & LANKELMA, J. (1987). Daunomycin accumulation in resistant tumor cells as a screening model for resistance modifiing drugs: role of protein binding. *Cancer Lett.*, **35**, 87-95.

BROXTERMAN, H.J., KUIPER, C.M., SCHUURHUIS, G.J., TSURUO, T., PINEDO, H.M. & LANKELMA, J. (1988). Increase of daunorubicin and vincristine accumulation in multidrug resistant human ovarian carcinoma cells by a monoclonal antibody reacting with P-glycoprotein. *Biochem. Pharmacol.*, **37**, 2389-2393.

BROXTERMAN, H.J., PINEDO, H.M., KUIPER, C.M., VAN DER HOEVEN, J.J.M., DE LANGE, P., BAAK, J.J., SCHEPER, R.J., KEIZER, H.G., SCHUURHUIS, G.J. & LANKELMA, J. (1989). Immunohistochemical detection of P-glycoprotein in human tumor cells with a low degree of drug resistance. *Int. J. Cancer*, **43**, 340-343.

CAMPLING, B.G., PYM, J., GALBRAITH, P.R. & COLE, S.P.C. (1988). Use of the MTT assay for rapid determination of chemosensitivity of human leukemic blast cells. *Leuk. Res.*, **12**, 823-831.

CARULLI, G., PETRINI, M., MARINI, A. & AMBROGI, F. (1988). P-glycoprotein in acute nonlymphoblastic leukemia and in the blastic crisis of myeloid leukemia. *N. Engl. J. Med.*, **319**, 797-798.

DALTON, W.S., GROGAN, T.M., RYBSKI, J.A., SCHEPER, R.J., RICHTER, L., KAILEY, J., BROXTERMAN, H.J., PINEDO, H.M. & SALMON, S.E. (1989). Immunohistochemical detection and quantitation of P-glycoprotein in multiple drug-resistant human myeloma cells: association with level of drug resistance and drug accumulation. *Blood*, **73**, 747-752.

EVANS, W.E., PETROS, W.P., RELLING, M.V., CROM, W.R., MADDEN, T., RODMAN, J.H. & SUNDERLAND, M. (1989). Clinical pharmacology of cancer chemotherapy in children. *Pediatr. Clin. North Am.*, **36**, 1199-1230.

HABER, M., NORRIS, M.D., KAVALLARIS, M., BELL, D.R., DAVEY, D.R., WHITE, L. & STEWART, B.W. (1989). Atypical multidrug resistance in a therapy-induced drug-resistant human leukemia cell line (LALW-2): resistance to vinca alkaloids independent of p-glycoprotein. *Cancer Res.*, **49**, 5281-5287.

HONGO, T., FUJII, Y., MIZUNO, Y., HARAGUCHI, S. & YOSHIDA, T.O. (1987). Anticancer drug sensitivity test using the short-term microplate culture and MTT dye reduction assay. *Jpn. J. Cancer Chemother.*, **14**, 472-478.

HONGO, T., FUJII, Y. & IGARASHI, Y. (1990). An *in vitro* chemosensitivity test for the screening of anti-cancer drugs in childhood leukemia. *Cancer*, **65**, 1263-1272.

KASPERS, G.J., PIETERS, R., VAN ZANTWIJK, C.H., DE LAAT, P.A.J.M., DE WAAL, F.C., VAN WERING, E.R. & VEERMAN, A.J.P. (1991). *In vitro* drug sensitivity of normal peripheral blood lymphocytes and childhood leukemic cells from bone marrow and peripheral blood. *Br. J. Cancer*, **64**, 469-474.

KLUMPER, E., PIETERS, R., KASPERS, G.J.L., VAN WERING, E.R., HÄHLEN, K. & VEERMAN, A.J.P. (1991). Cytostatic drug resistance in childhood relapsed acute lymphoblastic leukemia (ALL). 2nd Int Symposium on Cytostatic Drug Resistance, Kiel, Germany, 1-2 November 1991.

MA, D.D.F., DAVEY, R.A., HARMAN, D.H.,ISBISTER, J.P., SCURR, R.D., MACKERTICH, S.M., DOWDEN, G. & BELL, D.R. (1987). Detection of a multidrug resistant phenotype in acute nonlymphoblastic leukaemia. *Lancet*, **i**, 135-137.

MARUYAMA, Y., MUROHASHI, I., NARA, N. & AOKI, N. (1989). Effects of verapamil on the cellular accumulation of daunorubicin in blast cells and on the chemosensitivity of leukaemic blast progenitors in acute myelogenous leukaemia. *Br. J. Haematol.*, **72**, 357-362.

MATTERN, J., EFFERTH, T., BAK, M., HO, A.D. & VOLM, M. (1989). Detection of p-glycoprotein in human leukemias using monoclonal antibodies. *Blut*, **58**, 215-217.

PIETERS, R., HUISMANS, D.R., LEYVA, A. & VEERMAN, A.J.P. (1988). Adaptation of a rapid tetrazolium based (MTT-) assay for chemosensitivity testing in childhood leukemia. *Cancer Lett.*, **41**, 323-332.

PIETERS, R., HUISMANS, D.R., LEYVA, A. & VEERMAN, A.J.P. (1989). Comparison of the rapid automated MTT-assay with a dye exclusion assay for chemosensitivity testing in childhood leukemia. *Br. J. Cancer*, **59**, 217-220.

PIETERS, R., LOONEN, A.H., HUISMANS, D.R., BROEKEMA, G.J., DIRVEN, M.W.J., HEYENBROK, M.W., HÄHLEN, K. & VEERMAN, A.J.P. (1990). Detection of drug resistance in children with leukemia using the MTT assay with improved culture conditions. *Blood*, **76**, 2327-2336.

PIETERS, R., HUISMANS, D.R., LOONEN, A.H., HÄHLEN, K., VAN DER DOES-VAN DEN BERG, A., VAN WERING, E.R. & VEERMAN, A.J.P. (1991). Relation of cellular drug resistance to long-term clinical outcome in childhood acute lymphoblastic leukemia. *Lancet*, **338**, 399-403.

RIVERA, G.K., SANTANA, V., MAHMOUD, H., BUCHANAN, G. & CRIST, W.M. (1989). Acute lymphocytic leukemia of childhood: the problem of relapses. *Bone Marrow Transplant*, **4** suppl 1, 80-85.

RIVERA-FILLAT, M.P., PALLARES-TRUJILLO, J., DOMENECH, C. & GRAU-OLIETE, M.R. (1988). Comparative uptake, retention and action of vincristine, vinblastine and vindesine on murine leukaemic lymphoblasts sensitive and resistant to vincristine. *Br. J. Pharmacol.*, **93**, 902-908.

ROTHENBERG, M.L., MICKLEY, L.A., COLE, D.E., BALIS, F.M., TSURUO, T., POPLACK, D.G. & FOJO, A.T. (1989). Expression of the mdr-1/p-170 gene in patients with acute lymphoblastic leukemia. *Blood*, **74**: 1388-1395.

SANTINI, V., BERNABEI, P.A., SILVESTRO, L., DAL POZZO, O., BEZZINI, R., VIANO, I., GATTEI, V., SACCARDI, R. & ROSSI FERRINI, P. (1989). *In vitro* chemosensitivity testing of leukemic cells: prediction of response to chemotherapy in patients with acute non-lymphocytic leukemia. *Haematol. Oncol.*, **7**, 287-293.

SARGENT, J.M. & TAYLOR, C.G. (1989). Appraisal of the MTT assay as a rapid test of chemosensitivity in acute myeloid leukaemia. *Br. J. Cancer*, **60**, 206-210.

SCHUURHUIS, G.J., PINEDO, H.M., BROXTERMAN, H.J., VAN KALKEN, C.K., KUIPER, C.M. & LANKELMA, J. (1990). Differential sensitivity of multidrug resistant and sensitive cells to resistance modifiing agents and the relation with the reversal of anthracycline resistance. *Int. J. Cancer*, **46**, 330-336.

SUGAWARA, I., KODO, H., OHKOCHI, E., HAMADA, H., TSURUO, T. & MORI, S. (1989). High-level expression of MRK 16 and MRK 20 murine monoclonal antibody-defined proteins (170,000–180,000 P-glycoprotein and 85,000 protein) in leukaemias and malignant lymphomas. *Br. J. Cancer*, **60**, 538–541.

TAWA, A., ISHIHARA, S., YUMURA, K., HARA, J., INOUE, M., MURUYAMA, F., KAWAI, S., FUJIMOTO, T., NOBORI, U., NISHIKAWA, A., TSURUO, T. & KAWA-HA, K. (1990). Expression of the multidrug-resistance gene in childhood leukemia. *Jpn. J. Pediatr. Hematol.*, **4**, 38–43.

TIDEFELT, U., SUNDMAN-ENGELBERG, B. & PAUL, C. (1988). Effects of verapamil on uptake and *in vitro* toxicity of anthracyclines in human leukemic blastcells. *Eur. J. Haematol.*, **40**, 385–395.

TIDEFELT, U., SUNDMAN-ENGELBERG, B., RHEDIN, A.S. & PAUL, C. (1989). *In vitro* drug testing in patients with acute leukemia with incubations mimicking *in vivo* intracellular drug concentrations. *Eur. J. Haematol.*, **43**, 374–384.

TWENTYMAN, P.R., FOX, N.E. & REES, J.K.H. (1989). Chemosensitivity testing of fresh leukaemia cells using the MTT colorimetric assay. *Br. J. Haematol.*, **71**, 19–24.

UBEZIO, P., LIMONTA, M., D'INCALCI, M., DAMIA, G., MASERA, G., GIUDICI, G., WOLVERTO, J.S. & BECK, W.T. (1989). Failure to detect the P-glycoprotein multidrug resistant phenotype in cases of resistant childhood acute lymphocytic leukaemia. *Eur. J. Cancer Clin. Oncol.*, **25**, 1895.

VAN DIEST, P.J., SMEULDERS, A.W.M., THUNNISSEN, F.J.B.M. & BAAK, J.P.A. (1989). Cytophotometry: a methodologic study of preparation techniques, selection methods and sample sizes. *Analyt. Quant. Cytol. Histol.*, **11**, 225–231.

VEERMAN, A.J.P. & PIETERS, R. (1990). Drug sensitivity assays in leukaemia and lymphoma. *Br. J. Haematol.*, **74**, 381–384.

WEISENTHAL, L.M., DILL, P.L., FINKLESTEIN, J.Z., DUARTE, T.E., BAKER, J.A. & MORAN, E.M. (1986). Laboratory detection of primary and acquired drug resistance in human lymphatic neoplasms. *Cancer Treat. Rep.*, **70**, 1283–1295.

WEISENTHAL, L.M., SU, Y.-Z., DUARTE, T.E., DILL, P.L. & NAGOURNEY, R.A. (1987). Perturbation of *in vitro* drug resistance in human lymphatic neoplasms by combinations of putative inhibitors of protein kinase C. *Cancer Treat. Rep.*, **71**, 1239–1243.